# The Influence of Odors upon the Voice

**Published:** 1894-06

**Authors:** 


### The Influence of Odors upon the Voice.

You may, perchance, some day give a *soiree musicale,* as all fashionable people have the right to do, and you will be astonished to note how your singers will insist that no flowers shall be permitted to decorate your parlor; this will appear odd, too, when you first give the matter attention, and you will say to yourself: "How strange these musical people are!" But singers are not like the rest of the world; they seek to appear *singular* above all things. You might well say at the start, they are *originals.* They can only be compared to spoiled children, whose faults we pardon in view of the great pleasure they give us. That is the way the world and the average opera manager feels, and such criticisms are unjust and severe.

Flowers, and all perfumes in general, exercise an injurious influence on the vocal cords. There is no doubt about this fact, and, though rarely described, this peculiarity has been observed many, many times. What led us to study this question of the influence of odors upon the voice may be easily explained; *it was curiosity.* One day there fell under our eyes a "leaf" from a journal, on which was portrayed an etching. This person was an actress; her picture, like her name, need not be mentioned. She had a thousand good qualities and very few faults. We read the sketch of her life along with her etched face. This informed the public that she detested the odor of amber and musk and all other strong odors, and her listeners were warned not to perfume themselves when attending her concerts, because she believed, "according to Monnet Sully, that perfumes broke one's voice." —*The Cincinnati Lancet-Clinic.*

